# Recent Advances in Zwitterionic Hydrogels: Preparation, Property, and Biomedical Application

**DOI:** 10.3390/gels8010046

**Published:** 2022-01-07

**Authors:** Sihang Liu, Jingyi Tang, Fangqin Ji, Weifeng Lin, Shengfu Chen

**Affiliations:** 1 Key Laboratory of Biomass Chemical Engineering of Ministry of Education, College of Chemical and Biological Engineering, Zhejiang University, Hangzhou 310027, China; igorbarton@zju.edu.cn (S.L.); jytang@zju.edu.cn (J.T.); jifangqin@zju.edu.cn (F.J.); 2 State Key Laboratory of Advanced Optical Communication Systems and Networks, Key Laboratory for Thin Film and Microfabrication of the Ministry of Education, School of Electronic Information and Electrical Engineering, Shanghai Jiao Tong University, Shanghai 200240, China; 3 Zhejiang Development & Planning Institute, Hangzhou 310030, China; 4 Taizhou Technician College, Taizhou 318000, China; 5 Department of Molecular Chemistry and Materials Science, Weizmann Institute of Science, Rehovot 76100, Israel; 6 Key Laboratory of Biomedical Materials, College of Chemistry and Materials Science, Nanjing Normal University, Nanjing 210046, China

**Keywords:** antifouling materials, zwitterionic hydrogel, implantable devices, drug delivery, cellular therapy, cell culture, wound healing

## Abstract

Nonspecific protein adsorption impedes the sustainability of materials in biologically related applications. Such adsorption activates the immune system by quick identification of allogeneic materials and triggers a rejection, resulting in the rapid failure of implant materials and drugs. Antifouling materials have been rapidly developed in the past 20 years, from natural polysaccharides (such as dextran) to synthetic polymers (such as polyethylene glycol, PEG). However, recent studies have shown that traditional antifouling materials, including PEG, still fail to overcome the challenges of a complex human environment. Zwitterionic materials are a class of materials that contain both cationic and anionic groups, with their overall charge being neutral. Compared with PEG materials, zwitterionic materials have much stronger hydration, which is considered the most important factor for antifouling. Among zwitterionic materials, zwitterionic hydrogels have excellent structural stability and controllable regulation capabilities for various biomedical scenarios. Here, we first describe the mechanism and structure of zwitterionic materials. Following the preparation and property of zwitterionic hydrogels, recent advances in zwitterionic hydrogels in various biomedical applications are reviewed.

## 1. Introduction

Hydrogels are crosslinked (physically or chemically) hydrophilic polymer networks that retain large amounts of water, which are widely used in biocompatible implant devices, biosensors, drug delivery systems, wound care, and many other aspects due to their high biocompatible, tunable mechanical properties, and excellent permeability [1]. However, in the current research activities, the complexity of in vivo environments of hydrogel has not been fully considered. The objective existence of the biofouling phenomenon (protein adsorption, cell and bacterial adhesion, and biofilm development) has resulted in various adverse reactions such as decreased efficiency of implant equipment, immune rejection, and so on [2].

**Figure 1:**
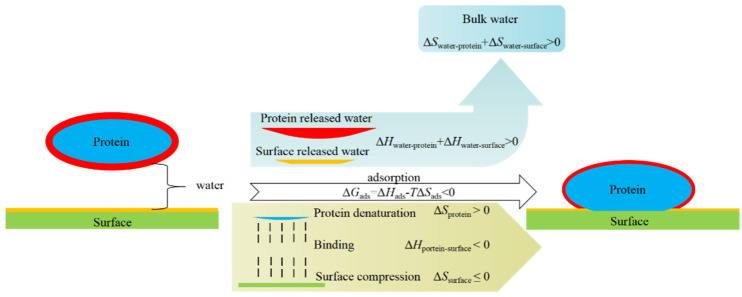
Scheme of mechanisms for nonspecific protein adsorption.

Biofouling comes from the inevitable interaction between protein molecules and material surface, including charge interactions (ion–ion interaction, ion–dipole interaction, dipole–dipole interaction, etc.), hydrogen bond interaction, hydrophobic interaction, Van der Waals force, and other more specific forces related to biological systems. However, in bioapplication environments, the coexistence of water molecules makes the interaction between protein and materials a ternary system. In the process of protein adsorption on the material surface, the bound water on the protein and material surface is released (Δ*H*_w__ater-p__rotein_ + Δ*H*_w__ater-s__urface_ > 0). The released water molecules change from a low degree of freedom (bound state) to a high degree of freedom (free state), with Δ*S*_w__ater-p__rotein_ + Δ*S*_w__ater-s__urface_ > 0. Then, the protein is adsorbed on the surface through multiple weak interactions (Δ*H*_p__rotein-s__urface_ < 0) and possible protein denaturation and surface compression occur during the adsorption process, which Δ*S*_p__rotein_ > 0 and Δ*S*_surface_ ≤ 0, respectively. Therefore, in the thermodynamics of protein adsorption, Δ*G*_ads_ = Δ*H*_ads_ − *T*Δ*S*_ads_ = (Δ*H*_w__ater-p__rotein_ + Δ*H*_w__ater-s__urface_ + Δ*H*_p__rotein-s__urface_) − *T*(Δ*S*_w__ater-p__rotein_ + Δ*S*_w__ater-s__urface_ + Δ*S*_p__rotein_ + Δ*S*_surface_). After the interaction between protein and surface is minimized, the Δ*H*_w__ater-s__urface_ is the decisive term for solid surfaces to resist biofouling (*T*Δ*S*_ads_ is always > 0) and *T*Δ*S*_surface_ also could make additional contributions to hydrophilic polymer-coated surfaces, and thus shows the synergistic effect to resist biofouling (Figure 1). However, it is a limited one when the protein molecules only stick to a polymer brush without compression [3]. Thus, the strong hydration of the surface, either solid surface or hydrophilic polymer-coated surfaces, is the key to resisting biofouling.

For materials with hydrophilic character, the surface of the material is firmly bonded with water molecules through hydrogen bonds. Thus, Δ*H*_water-surface_ increases when bound water molecules are released, which leads to the fouling process being unfavorable and shows an antifouling potential [4]. Among these hydrophilic polymers (such as PEG, poly(2-hydroxyethyl methacrylate) (pHEMA), and dextran), PEG has been regarded as the gold standard for antifouling materials due to its weaker interaction with proteins and efficient H-bond hydration [5]. However, in recent years, it is found that PEG cannot be regarded as the most ideal antifouling material. Firstly, the antifouling properties of PEG materials are strictly limited to the application environments [6,7]. PEG is vulnerable to oxidation under biological medium, which causes considerable protein adsorption [8]. Secondly, PEG materials still interact with proteins weakly [9,10]. Shao et al. [11] studied the influences of zwitterionic carboxybetaine (CB) moieties and non-ionic oligoethylene glycol (OEG) moieties on hydrophobic interaction of two nonpolar plates using well-tempered metadynamics simulations. Results showed that the hydrophobic interaction between two plates was the same in both the CB solution and water while those were weakened in OEG solution, and a new association state was observed, which means that OEG has a hydrophobic characteristic to disturb the hydrophobic interaction between two non-polar plates. Recent studies have also confirmed the existence of PEG antibodies, while zwitterionic materials exhibited minimal immunogenicity [12,13,14]. The existence of the above problems puts forward higher requirements for more effective antifouling materials.

**Figure 2:**
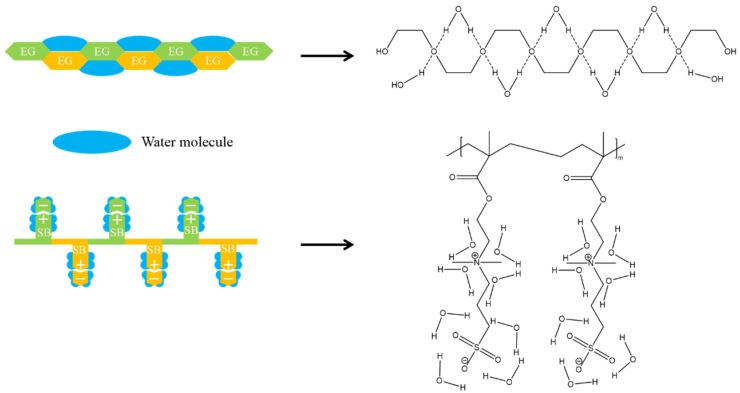
Tightly bonded hydration shell on zwitterionic polysulfobetaine (polySB) and PEG. Each EG unit binds 1 water molecule, while each SB unit binds 7–8 water molecules. Reproduced with permission from [17]. Copyright 2012, American Chemical Society.

Inspired by the excellent antifouling properties of zwitterionic phosphatidylcholine (a major component in the cell membrane), zwitterionic polymers have attracted much attention [15]. Compared with PEG-derived materials, zwitterionic materials have much stronger hydrophilicity due to their ionic solvation nature [16], with no hydrogen bond interaction or hydrophobic interaction with protein [11], which further increases the difference between Δ*H* and *T*Δ*S*. For example, polysulfobetaine (pSBMA) can bind 7–8 water molecules on each sulfobetaine (SB) unit through a stronger binding force than PEG, while the latter can only bind one water molecule on each EG unit (Figure 2) [17,18]. This stronger binding force increases the amount of Δ*H*_water-surface_ required for proteins to disrupt the surface hydration layer [19].

On the other hand, water molecules bonded on the zwitterionic surface have various orientations, while water molecules on the PEG surface tend to be directional [20]. Therefore, the bound water on the surface of zwitterions is more similar to bulk water and has higher degrees of freedom (increased initial *S* to decrease Δ*S*). These hydration characteristics make it more difficult for protein molecules to break through the hydration layer on the surface of the material, which is beneficial to the realization of resisting nonspecific protein adsorption (Δ*H* > *T*Δ*S*, Δ*G* > 0). In this review, we will introduce the properties of zwitterionic materials and summarize their progress in hydrogel applications.

## 2. Structure of Zwitterionic Materials

**Figure 3:**
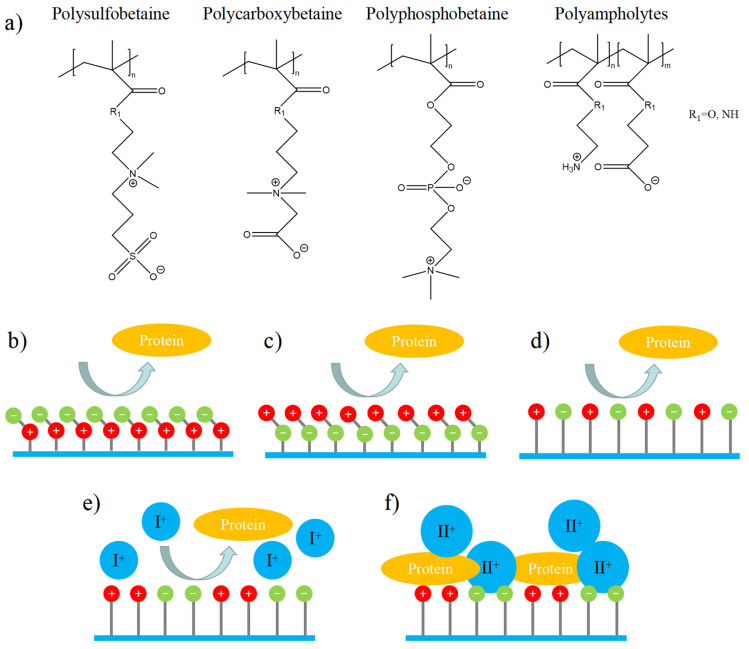
Schematic illustration of common zwitterionic polymers and their antifouling properties. (**a**) Structure of zwitterionic polymers. (**b**) Antifouling property of polybetaine with an anionic terminal group. (**c**) Antifouling property of polyphosphorylcholine with cationic terminal group. (**d**) Antifouling property of polyampholytes with strictly alternating charges. (**e**) Antifouling property of polyampholytes with continuously arranged charge in a monovalent ionic solution. (**f**) Antifouling property of polyampholytes with continuously arranged charge in a multivalent ionic solution.

Zwitterionic materials are a class of materials that contain both cationic and anionic groups, with no overall charge (neutral). Zwitterionic materials can be divided into the following categories according to the monomer structural characteristics (as shown in Figure 3a): (1) phosphobetaine with a phosphate group and a terminal quaternary ammonium group [21,22,23,24]; (2) sulfobetaine or carboxybetaine with a quaternary ammonium group and a terminal carboxyl or sulfonic group [25,26,27,28]; (3) zwitterionic polyampholytes [29]. For the first two kinds of materials, each side-chain unit in the polymer contains an equal amount of positive and negative charges, which is electrically neutral. There is no/low interaction between the polymer and protein, which can have a stable and long-term antifouling effect in a variety of complex environments (Figure 3b–d). For the last one, the polymer chain is electrically neutral as a whole, and whether the positive and negative charges are evenly distributed or not has a significant impact on its antifouling properties (Figure 3e,f) [30].

For example, in a monovalent ionic solution, zwitterionic peptides with zwitterionic EKEKEKEK (K: lysine; E: glutamic acid) fragment or EEEEKKKK both showed antifouling property, while in a multivalent ionic solution, only peptides with EKEKEKEK showed antifouling. This is since the multivalent cations are easy to chelate with the anionic functional groups, which makes the surface of the material lose the structural characteristics of zwitterions [30]. Therefore, in the following introduction of this paper, we will focus on phosphobetaine, sulfobetainebetaine, carboxybetaine, and EK polypeptides or polyaminoacids with uniform charge distribution, without further expansion of other synthetic polyampholytes.

## 3. Zwitterionic Hydrogels

### 3.1. Preparation of Zwitterionic Hydrogels

**Figure 4:**
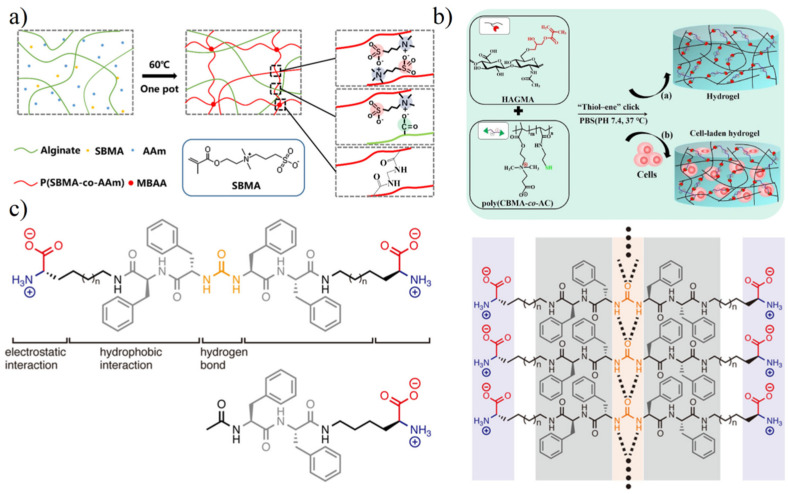
Methods for the formation of zwitterionic hydrogels. (**a**) In situ free radical polymerization using SBMA and acrylamide (AAm) monomers initiated by heat. Reproduced with permission from [44]. Copyright 2020 Wiley Periodicals LLC. (**b**) Polymer-coupling using thiol-ene click reaction without small molecules. Reproduced with permission from [36]. Copyright 2020 Elsevier Ltd. All rights reserved. (**c**) Self-assembly using urea bond to form a strong hydrogen bond network. Reproduced with permission from [42]. Copyright 2020 Wiley Periodicals LLC.

The preparation of zwitterionic hydrogels is mainly achieved by the following means (as shown in Figure 4): (1) radical polymerization; (2) polymer coupling; (3) self-assembly through physical interactions (supramolecular hydrogel).

Free radical polymerization is easy to operate. Zwitterionic monomers (methacrylate/acrylate/methacrylamide/acrylamide) grow through light, heat, or redox agents [31] and combine with the crosslinking agents to achieve the gelation (Figure 4a). Traditional chemical crosslinking agents (non-zwitterionic agents, such as *N*,*N*′-methylenebisacrylamide, MBAA) may potentially damage the antifouling property of zwitterionic hydrogel in complex biological environments [32]. Dimethacrylated zwitterionic crosslinkers become necessary agents to form zwitterionic hydrogels by radical polymerization for environments that require extremely high antifouling performance [33]. However, based on practical application, hydrogels prepared by free radical polymerization often have unreactive monomers, which can diffuse into the biological medium during the applications and then harm bio-safety. The complete removal of unreacted monomers often requires multiple solvent extraction steps, which is extremely detrimental to the continuous production.

Polymer coupling requires appropriate small molecules for gelation. Coupling agent 1-Ethyl-3-(3-dimethylaminopropyl) carbodiimide·HCl (EDC·HCl) is suitable to prepare zwitterionic peptide EK hydrogel via coupling reaction between carboxyl groups and primary amines [34,35]. However, the residual EDC molecules are still biohazard components. Click reaction is relatively enough to provide a cleaner environment. For example, in order to better analyze the behavior and influencing factors in the cell growth process, the hydrogels for cell culture are very strict on potential small molecules. Hydrogel prepared by thiol-ene [36] (Figure 4b) or strain-promoted azide–alkyne [37] click reaction can avoid the residue of small molecules as much as possible to reduce the background noise of the 3D cell growth process.

Self-assembly supramolecular hydrogels were prepared by a strong physical interaction. Both amino acid derivatives and dipeptides have been recognized as supramolecular hydrogelator in recent years [38,39]. Hydrophobic interaction or hydrogen bond acts as a physical crosslinker in self-assembly peptide hydrogels. Phenylalanine peptide and its derivatives are suitable for self-assembly prepared transparent hydrogel due to a strong π-π stacking on aromatic groups [40,41]. Tsutsumi et al. [42] further introduced a urea bond to form a strong hydrogen bond network to stabilize the self-assembly structure (Figure 4c). There are no potential toxic small molecules retained during hydrogel formation in this approach, which is suitable for the clean environment required application [42,43].

### 3.2. Properties of Zwitterionic Hydrogels

As a soft material, the properties of hydrogels determine their potential application. Therefore, before introducing the application of zwitterionic hydrogels, we need to review the inherent properties of zwitterionic hydrogels themselves (other than antifouling properties) so as to correlate the subsequent application scenarios.

#### 3.2.1. Swelling Behavior and Mechanical Properties

Although zwitterions have strong electrolyte functional groups, zwitterionic hydrogels behave quite differently from polyelectrolyte hydrogels in salt solutions [45]. In pure water, a strong dipole–dipole interaction between the polymer side-chains in the zwitterionic hydrogel is observed [46]. The network of hydrogels is not only controlled by crosslinkers but also by a large number of dipole–dipole interactions in pure water [47]. In contrast, the dipole–dipole interaction in zwitterionic hydrogels is destroyed by adding salts, which decreases the density of the zwitterionic hydrogel network and swells zwitterionic hydrogels (so-called anti-polyelectrolytes effect).

The mechanical strength of zwitterionic hydrogel is related to crosslinking network density and water content. In pure water, a zwitterionic hydrogel with low crosslink density (less than 1 mol%) is relatively flexible, showing low elastic modulus and high strain. However, in a salt solution, the further swelling due to the anti-polyelectrolyte effect causes the hydrogel to become extremely fragile [48]. As an ideal candidate for bioengineering materials, designing zwitterionic hydrogels that meet mechanical strength requirements is a prerequisite for practical applications.

The most commonly used method to regulate the anti-polyelectrolytes effect of zwitterionic hydrogels is to copolymerize other types of monomers with zwitterionic ions. The copolymerization of electrolyte monomers (such as acrylic acid, AA) or neutral monomers (such as N-isopropyl acrylamide, NIPAM) can effectively suppress the anti-polyelectrolytes effects of zwitterionic hydrogels [49]. However, the high molar ratio of these monomers to zwitterionic monomers (more than 1:1) seriously damages antifouling property. The copolymerization of hydrophobic groups exhibited less damage to antifouling property due to the fewer hydrophobic monomers required to be incorporated into hydrogels for regulating the anti-polyelectrolytes effect. Hydrogel with sodium p-styrene sulfonate (NaSS) ([NaSS]/[SBMA] = 0.5) showed 0.41 in *SR*_equilibrium, NaCl_/*SR*_equilibrium, water_ (Table 1) [49]. In which SR is the equilibrium swelling ratio of the hydrogels can be calculated based on the weights of wet and dry hydrogels by SR = (W_e_ − W_d_)/W_d_. The hydrophobic interaction between aromatic groups was strongly enhanced in a salt solution, which indicated that zwitterionic hydrogels prepared with quite a few NaSS showed neither shrink nor swelling.

**Figure 5:**
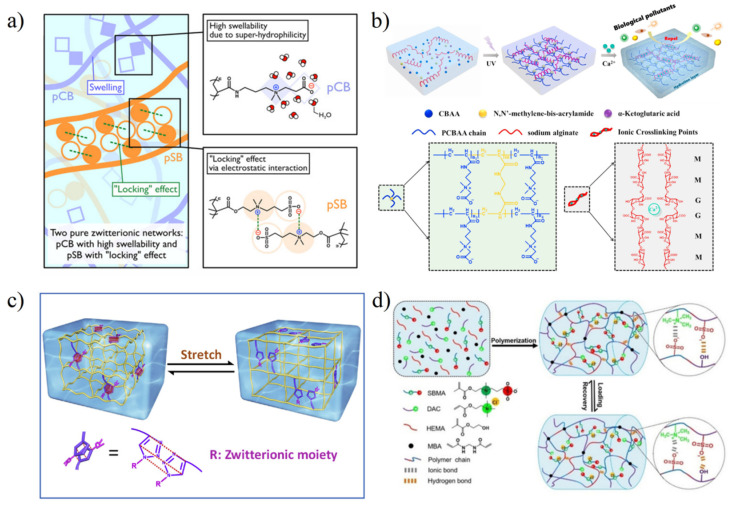
Methods to strengthen zwitterionic hydrogel. (**a**) Formation of zwitterionic-elastic-network (ZEN) with 1st PCBAA network and 2nd PSBMA network. Strong dipole–dipole interaction between SB units acted as physical crosslinking joints during load exertion to strengthen hydrogel. Reproduced with permission from [47]. Copyright 2021 The Authors, some rights reserved; exclusive licensee American Association for the Advancement of Science. (**b**) Preparation of double network (DN) with alginate-Ca^2+^ network and PCBAA network. Reproduced with permission from [51]. Copyright 2021 Elsevier Ltd. All rights reserved. (**c**) Zwitterionic molecule redesign with the π-π stacking between the triazole rings to strengthen hydrogel. Reproduced with permission from [57]. Copyright 2019 Elsevier Ltd. All rights reserved. (**d**) Copolymerization of SBMA, cationic monomer (DAC), and HEMA with reversible ionic bonds and hydrogen bonds to strengthen hydrogel. Reproduced with permission from [60]. Copyright 2020 Wiley-VCH GmbH.

For pure zwitterionic hydrogel, the dipole–dipole interaction of the side-chain can be the basis of the strengthened network in the stress-loading process by transferring mechanical energy to heat energy via the formation and destruction of physical crosslinking joints [11,47]. However, compared with hydrophobic interaction, hydrogen bonding interaction, π-π stacking, and other interactions, the dipole–dipole interaction is relatively weak due to a large number of hydrated water molecules on zwitterionic groups. Zwitterionic hydrogels are often destroyed by stress before realizing dipole–dipole strengthening. According to the analysis of the molecular structure of zwitterions by Shao et al. [11], pSBMA hydrogel is more easily strengthened by dipole–dipole interaction, which is attributed to the balanced charge density between anion and cation. PCBMA-pSBMA zwitterionic elastomers hydrogel prepared by Dong et al. (Figure 5a) [47], and polyurethane electrospun-pSBMA composite hydrogels prepared by Liu et al. [50] enhanced the dipole–dipole interaction of the SB units by applying additional stress to the pSBMA network, which further enhanced the mechanical strength of zwitterionic hydrogels. In addition, using pSBMA or poly (carboxybetaine acrylamide) (pCBAA) and natural polymers such as alginate to prepare double network hydrogels is also the main method to enhance the mechanical properties of zwitterionic hydrogels (Figure 5b) [51].

Recently, pure zwitterionic triple-network (ZTN) hydrogel was prepared to achieve high mechanical strength, excellent antifouling property, and overcome the anti-polyelectrolyte effect [52]. The poly (trimethylamine N-oxide) (PTMAO), a biomimetic polymer inspired by seawater fish, was used as the first network with high swelling properties. The second and third networks were formed using PSBMA with strong electrostatic interaction and network entanglement. ZTN hydrogel showed nearly 20 MPa compressive stress and 99% compressive strain in both PBS and seawater. The SR_equibrium, seawater_ of ZTN hydrogel was only increased from 150% (SR_equibrium, water_) to 300%, which indicated that the strong salt swelling caused by the anti-electrolyte effect is successfully suppressed.

It is worth noting that in the design of ordinary double network hydrogels, the second networks composed of flexible polymers often do not add crosslinking agents in the process of preparation but interact with the first network through physical action. However, for zwitterionic polymer, due to the strong hydration of its molecular surface, if the zwitterionic polymer network is not stabilized by crosslinker in a salt solution, the antifouling zwitterionic network will release slowly, resulting in the loss of functionality [6,44,47,50,53].

On the premise of keeping enough antifouling properties, other functional groups which can be used to stabilize polymer networks are also gradually introduced to enhance zwitterionic hydrogels. There are two main categories: (1) introducing hydrophobic groups/intermolecular hydrogen-bond formation moieties into zwitterionic side-chain (Figure 5c) [54,55,56,57]; (2) copolymerization of zwitterionic monomers with different functional monomers (Figure 5d) [44,58,59,60,61,62,63,64,65,66]. At present, through the selection of different monomers, zwitterionic hydrogels have been able to achieve various aspects of strength and elasticity regulation to promote their application.

#### 3.2.2. Self-Healing

**Figure 6:**
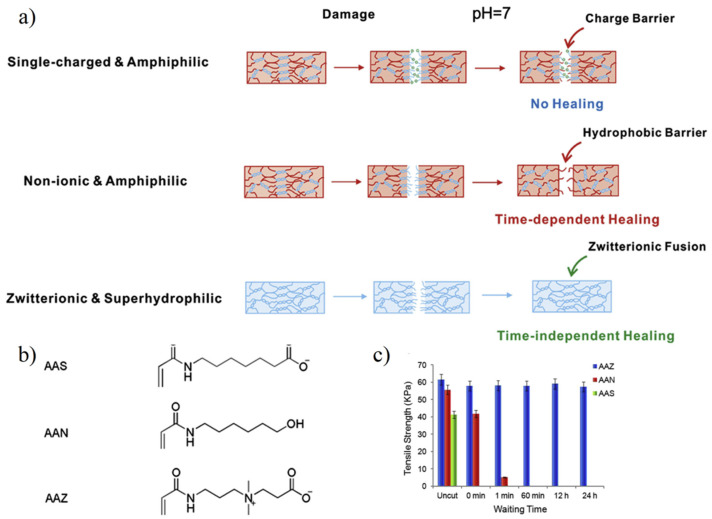
Zwitterionic fusion induced time-independent self-healing. (**a**) Healing behaviors of single-charged, non-ionic, and zwitterionic hydrogels. For hydrogels prepared by single-charged monomers, charge barrier hindered self-healing on neutral conditions. For hydrogels prepared by non-ionic monomers, hydrophobic barrier formation in a short time, which determined potential time interval to self-healing. (**b**) Chemical structure of single-charged anionic 7-acrylamidoheptanoate acid (AAS), non-ionic hydroxyhexylacrylamide (AAN), and zwitterionic CBAA (AAZ). (**c**) The tensile strength of different hydrogels. AAS hydrogels fractured and showed no self-healing behavior. AAN hydrogels could only self-healing after being cut within 1 min. AAZ hydrogels self-healed and kept original tensile strength even the cut pieces were separated for 24 h. Reproduced with permission from [69]. Copyright 2014 Elsevier Ltd. All rights reserved.

The self-healing capability of hydrogel determines whether it can be applied in a harsh environment [67,68]. Improving the service life of hydrogels can effectively reduce costs and enhance the sustainability of materials used. Zwitterionic hydrogel has the ability of time-independent self-healing, which is also called zwitterionic fusion [69,70]. When destroyed, zwitterionic hydrogel surface can quickly combine with air–water, forming a long-time stable water layer, ensuring that hydrophilic functional groups still exist outside the section. When the zwitterionic hydrogel fragments are in contact with each other, the cationic and anionic functional groups of zwitterionic materials can be rapidly fused to form zwitterionic pairs via electrostatic attraction, thereby achieving self-healing. Bai et al. [69] compared the self-healing properties of zwitterionic carboxylic acid betaine acrylamide hydrogel and non-ionic hydrogel. Results showed that the zwitterionic hydrogel retained a self-healing ability after being cut 24 h (Figure 6), but the non-ionic polymer lost its self-healing ability after 1 min due to the hydrophobic reconstruction at the cutting surface. Sinclair et al. [70] used the zwitterion fusion characteristics and, based on the difference in the number of carbon atoms between charged functional groups, designed zwitterionic injectable pellets (ZIP) PCB-1 with better softness and suitable for cell culture and ZIP PCB-2 with better elasticity and suitable for drug delivery.

In addition, zwitterionic copolymers with other monomers, using zwitterionic fusion and other spontaneous repair methods to prepare self-healing hydrogels, are also effective ways to improve the lifetime of hydrogels. For example, Lin et al. [71] prepared poly(*N*,*N*-dimethyl-N(3-methacrylamidopropyl)-*N*-(3-sulfopropyl) ammonium betaine (DMAPMAPS)-HEMA) hydrogel with laponite XLG physical crosslinker. The synergistic effect of polymer-clay interaction and zwitterionic fusion provides self-healing ability for hydrogels.

#### 3.2.3. Adhesion and Lubricity

**Figure 7:**
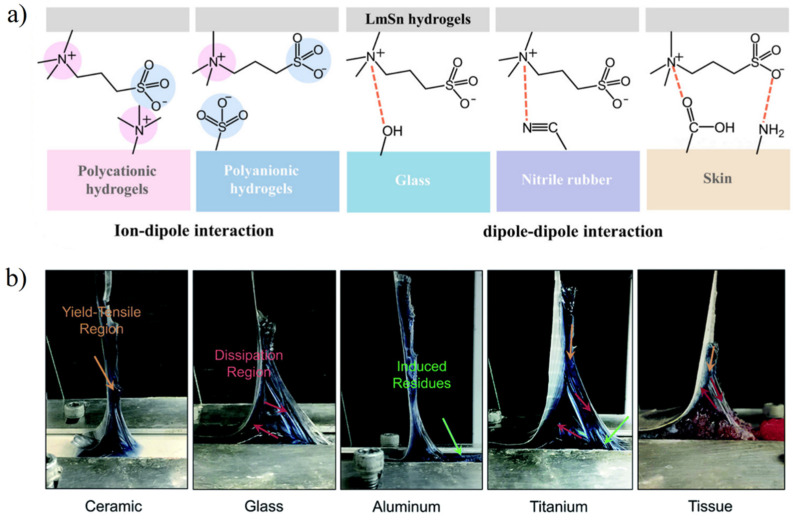
Adhesion between zwitterionic hydrogels and various substrates. (**a**) Adhesive mechanism of Laponite XLG composite SBMA hydrogel (LmSn) via ion–dipole interaction and dipole–dipole interaction between hydrogel and surface. Reproduced with permission from [74]. Copyright 2019, American Chemical Society. (**b**) Images of peeling off a test of adhesive zwitterionic hydrogel from nonporous surfaces. Synergistic of hydrogen bond interaction and dipolar interaction provided strong adhesive force. Reproduced with permission from [59]. Copyright 2020 The Royal Society of Chemistry.

The interfacial interaction between the hydrogel and other materials can be divided into two aspects: adhesion and lubrication. For zwitterionic hydrogels, the dipolar polymer side-chains can interact with other dipolar functional groups [72] and ionic functional groups [60,73] that may exist on the interface, thus showing adhesion properties. Zwitterions containing quaternary ammonium functional groups and sulfonic functional groups have strong dipole moments, which can form dipole–dipole interactions with polar functional groups such as carboxyl and amino groups in the skin and tissues and achieve the adhesion between hydrogels and tissues (Figure 7a) [63,74]. However, dipole–dipole interaction and dipole–ion interaction are difficult to form when the interface between the zwitterionic hydrogel and contacted surfaces has a strong hydration layer. Although the copolymerization of monomers with hydrogen bonding [59] (Figure 7b) or electrostatic interaction [73] can improve the adhesion of zwitterionic hydrogels, the catechol functional group is still most widely used in hydrogel adhesives due to the much stronger hydrogen bonding, electrostatic interaction, and π−π stacking in water [75]. The copolymerization of methacrylated dopamine and zwitterionic methacrylate monomer can significantly improve the limitations of zwitterionic hydrogels applied to wet surfaces [63,76].

**Figure 8:**
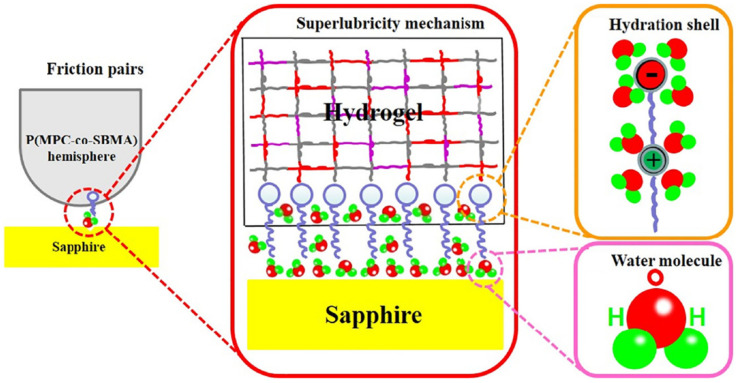
Superlubricity mechanism between P(MPC-co-SBMA) copolymer hydrogel and sapphire. Stabilized hydration layer formation contributed by strong hydration effect of zwitterionic MPC and strong adsorption of water on the sapphire surface. Reproduced with permission from [80]. Copyright 2019 The Authors. Published by Elsevier Ltd.

Although the strong hydration of the zwitterionic side-chain hinders the formation of the dipole–dipole interaction or dipole–ion interaction between the zwitterionic hydrogel and contact surface, it also means zwitterionic hydrogels could show excellent lubrication behavior based on the hydration lubrication mechanism [77,78,79]. Methacrylated phosphobetaine (MPC)-based copolymers reduce the slide friction substantially due to the high lubricity of MPC units, which have become a major method to synthesis lubricated hydrogels [77]. Wang et al. [80,81] prepared poly (MPC-co-SBMA) showed superlubricity (COF ~ 0.002) at sliding velocity of 48 mm/s and applied load of 4 N in water. The introduction of zwitterionic polymer increased the water-binding property, and the water molecules strongly adsorbed on the sapphire–hydrogel interface to form stabilized hydration layer, which sufficiently avoided direct asperity contact during sliding (Figure 8).

#### 3.2.4. High Ionic Conductivity

**Figure 9:**
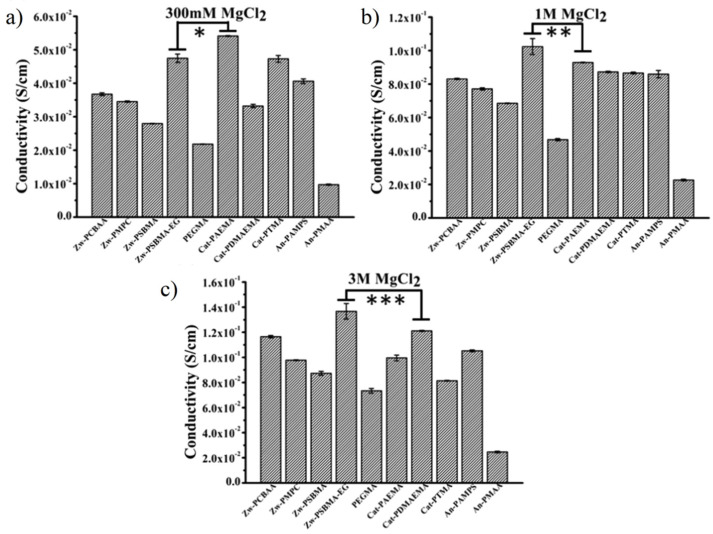
Ion conductivity of neutral and ionic hydrogel in MgCl_2_ solution with different concentration: (**a**) 300 mM, (**b**) 1 M and (**c**) 3M. *, **, *** *p* < 0.05. Zwitterionic hydrogel Zw-PSBMA-EG showed higher conductivity than both cationic hydrogel Cat-PAEMA and anionic hydrogel An-PAMPS in more than 1 M MgCl_2_ solution. Reproduced with permission from [83]. Copyright 2018, American Chemical Society.

Ionic conductivity is an important property for the application of biosensors and bioelectronics. Zwitterions showed more effective separation of cationic and anionic counterions during ion migration [82]. Compared to cationic and anionic polyelectrolyte hydrogels, zwitterionic hydrogels showed higher ionic conductivity in high concentrations of saline solutions [83,84]. Although all the polyelectrolyte hydrogels achieve better ionic conductivity with the increasing of the concentration of the saline solution, zwitterionic hydrogels showed a much higher increasing rate, in which the ionic conductivity of Zw-SBMA-EG hydrogel surpassed both cationic and anionic hydrogels in over 1 M MgCl_2_ solution (Figure 9) [83].

## 4. Biomedical Applications of Zwitterionic Hydrogels

**Figure 10:**
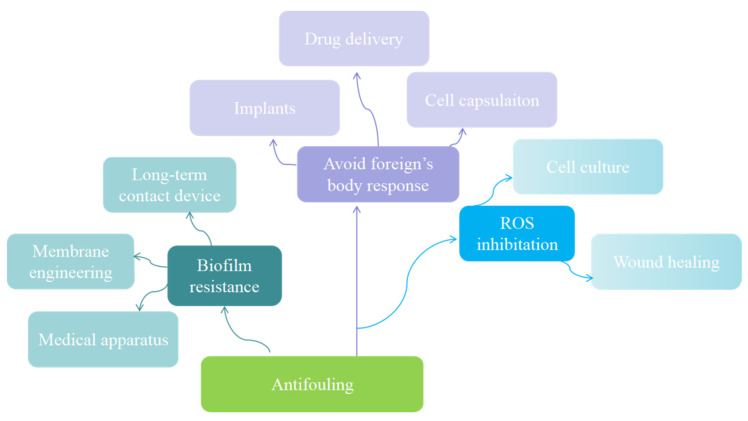
Application of antifouling zwitterionic hydrogel.

Antifouling ability is the foundation of hydrogel in the field of biological contact applications. From basic pollution avoidance to avoidance of immune system recognition, zwitterionic hydrogels have made considerable research achievements in engineering and medical fields (Figure 10).

### 4.1. Antifouling Coatings for Reusable Medical Apparatus in Physiological Environment

Biofouling on reusable medical apparatus in physiological environments (such as an endoscope, surgical equipment, contact lenses, etc.) has a more serious impact on the materials [85]. Biofouling phenomenon leads to the formation of bacterial microbial membrane, which has the potential risk of local infection. Furthermore, the biofouling base membrane [86] will become the medium for the reproduction of bacteria and other microorganisms and then produce biofilm [87], and then more organisms begin to grow [88]. Cleaning with bactericides and surfactants is a conventional treatment method for reusable medical devices. However, infections have still become an important safety hazard with the bacteria growth [89]. Therefore, the combination of effective cleaning and construction of anti-bacteria adhesive coating is necessary to reduce infection risk. The antifouling modified surface can resist not only nonspecific protein adsorption but also bacteria adhesion, [90] cell adhesion, [91] and platelet adhesion [92]. Liu et al. [93] prepared poly (carboxybetaine-co-dopamine methylamide) (pCBDA) copolymer and crosslinked it with a small amount of Cu^2+^ to modify the contact lens. The study confirmed that the protein adsorption of PCBDA hydrogel coating under 12 h and 24 h was 1.3 and 1.5 μg/cm^2^, respectively, and it resisted pathogenic microorganisms killed by Cu^2+^ ions. Recently, Wu et al. [94] designed a triple-layer patch with an antifouling polysulfobetaines layer to resist bacteria adhesion, fibrinogen adsorption, and fibrin capsule formation. The fabrication strategy based on origami made the patch be readily integrated with a variety of minimally invasive end effectors.

Another effective strategy for reusable medical apparatus is to design smart zwitterionic polymer with tunable bactericidal properties and anti-bacterial adhesion. Cao et al. [95] used the hydrolysis characteristics of N, N-dimethyl-2-morpholinone derivatives to prepare a coating for the conversion between hydroxylated zwitterionic carboxybetaine (CB-OH) and cyclic cations (CB-Ring). Results showed under dry conditions, the surface of the CB ring killed more than 99.9% of *Escherichia coli* (*E. coli*) K12 attached to it, while in a neutral or alkaline water environment, CB-Ring is immediately hydrolyzed to CB-OH then resists bacterial adhesion in an aqueous medium. Cao et al. [96] prepared poly(2-((2hydroxyethyl)(2-(methacryloyloxy)ethyl)(methyl) ammonio) acetate) (pCBOH1) and poly(2-(bis(2-hydroxyethyl)(2-(methacryloyloxy)ethyl) ammonio)acetate) (pCBOH2) hydrogels as novel materials for both bactericidal properties and anti-bacterial adhesion. Huang et al. [97] prepared salt responsive interpenetrating network (IPN) hydrogel with cationic poly((13rimethylamine)ethyl methacrylate chloride) (pTMAEMA) and zwitterionic poly(sulfobetaine vinylimidazole) (pSBVI). The prepared hydrogel showed a high bacterial killing rate after incubating IPN hydrogel in *Staphylococcus epidermidis* (*S. epidermidis*) and *E. coli*. Moreover, after washing in 1.0 M NaCl solution, IPN hydrogels released more than 96% bacteria.

**Figure 11:**
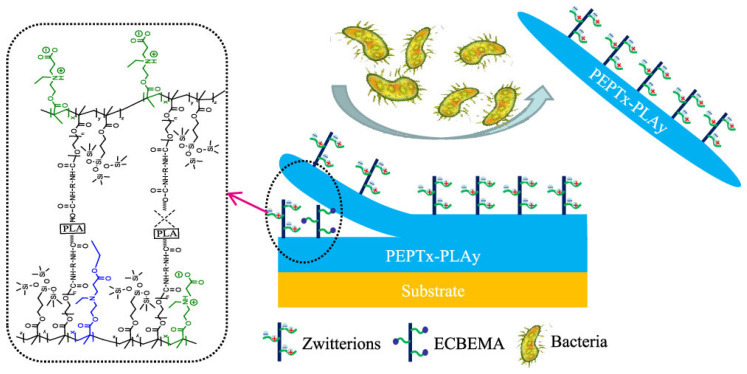
Long-term antifouling PCB-ester-PU with degradable IPDI-PLA-IPDI crosslinker. Reproduced with permission from [101]. Copyright 2020, American Chemical Society.

However, as long-term antifouling material, the hydrogel coating is still very vulnerable. Antifouling performance may be lost due to peeling or scratching caused by physical impact or degradation in the aqueous active environment (such as water, oxygen, and possible catalytic ions). Lin et al. [98] designed hydrophobic zwitterionic precursor-based material, which could improve mechanic strength and slow the degradation by increasing the hydrophobicity of polymer matrix and reducing the contact between the polymer matrix with reactive mediums. Ji et al. [99] combined hydrophobic CB-ester with medical gauze for long-term thrombosis resistance in blood. Wang et al. [100] prepared hydrogel with polyurethane (PU) main chains and CB-ester side-chains (PCB-ester-PU). Results showed PCB-ester-PU exhibited excellent resistance to nonspecific protein adsorption in short-time hydrolysis and maintained over 30 days in PBS. Ma et al. [101] further used isocyanate-terminated polylactic acid (IPDI-PLA-IPDI) as a degradable crosslinker in the PCB-ester-PU hydrogel to achieve self-regenerated antifouling property (Figure 11).

### 4.2. Membrane Separation Technology

**Figure 12:**
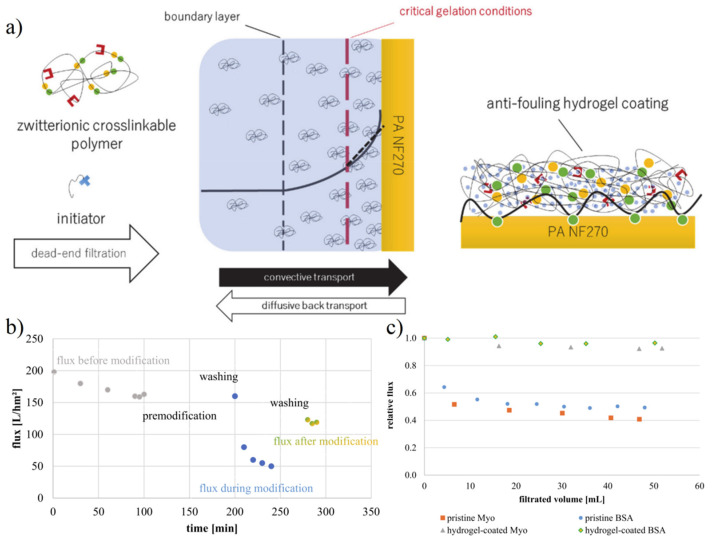
Antifouling zwitterionic hydrogel on the membrane to resist flux loss caused by nonspecific protein adsorption. (**a**) Schematic illustration of zwitterionic hydrogel coating on nanofiltration membrane via concentration polarization. (**b**) A dense hydrogel network caused membrane flux loss. (**c**) The relative flux was retained in the protein solution. Reproduced with permission from [27]. Copyright 2021 MDPI.

Membrane separation technology is an important technology to realize the separation of different components in the industry [102]. However, the cost of membrane separation is still high compared with other separation methods, which largely lies in the decline of efficiency caused by biofouling in the process of membrane use [103,104]. The biofouling problem will lead to the blockage of membrane pores and the formation of biolayer on the membrane surface, increasing the resistance of substances passing through the membrane and reducing the membrane flux [105]. Whether the membrane is regenerated under strict conditions or directly replaced, the cost will be increased. The zwitterionic coating can avoid the accumulation of biofouling substances on the membrane surface, minimize pore blockage and biolayer formation to maintain the membrane flux [23,106,107,108]. Quilitzsch et al. [104] prepared copolymers of 2-(dimethylamino) ethyl methacrylate (DMAEMA) and BMA, which can be adhered to the surface of the PES membrane by using hydrophobic interaction as a co-initiator layer. Subsequently, the hydrogel thin layer was prepared by using [3-(methacryloylamino) propyl] dimethyl (3-sulfopropyl) ammonium hydroxide (SBAA) via in situ redox polymerization. Freger et al. [109] modified the membrane by concentration polarization induced hydrogel free radical polymerization. Based on concentration polarization, May et al. [27] and Laghmari et al. [107] prepared zwitterionic SBMA copolymers containing activated carbon double bonds, which further improved the gelation state of hydrogels (Figure 12).

Venault et al. [110] used octadecyl acrylate and DMAEMA for copolymerization and then used 3-iodopropionic acid to achieve zwitterionic copolymer. The zwitterionic copolymer realizes the interaction with the polypropylene (PP) membrane and the crosslinking of the hydrogel thin layer by hydrophobic interaction of the octadecyl group. Under the coating density of 0.2 mg/cm^2^, the zwitterionic hydrogel thin layer modified PP film can maintain excellent antifouling performance. Various types of blood cells were isolated in the blood filtration experiment, and the activated proportion of filtered platelets also decreased significantly.

### 4.3. Antifouling Probe for Sensitive Detection

**Figure 13:**
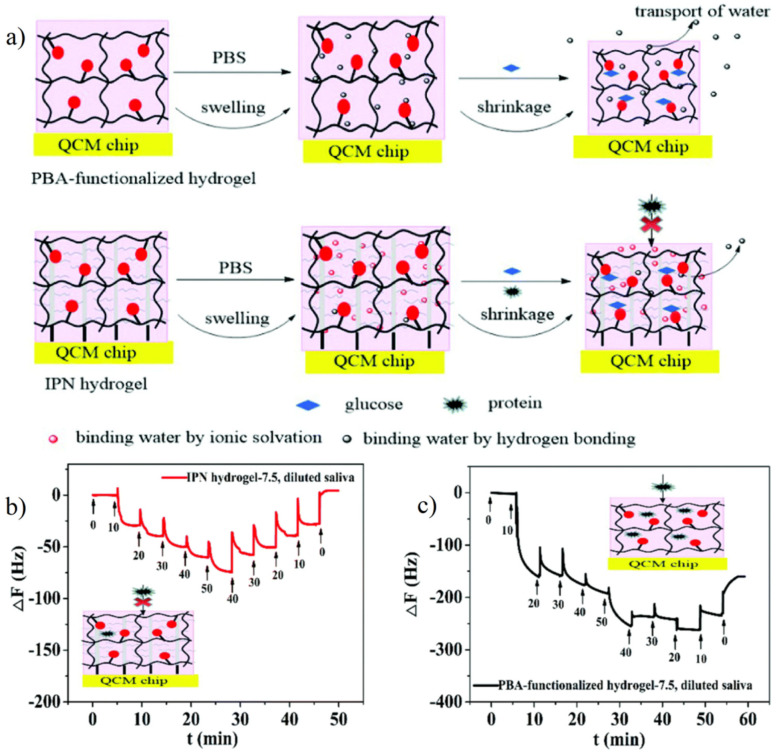
Zwitterionic interpenetrating network (IPN) hydrogel coating on QCM probe for highly sensitive glucose detection. (**a**) Schematic illustration of antifouling property of zwitterionic IPN hydrogel with glucose detection by PBA. (**b**,**c**) Glucose detection in 10% diluted saliva on zwitterionic IPN hydrogel coating QCM (**b**) and only PBA hydrogel coating QCM only (**c**). During the detection process with increased glucose concentration (10 to 50 mg/L), the frequency shift of IPN hydrogel coating QCM increased with the concentration increasing while those of PBA hydrogel coating QCM only showed a great increase in 10 mg/L. During the detection process with decreased glucose concentration, the only frequency shift of IPN hydrogel coating QCM also showed concentration correlation. These differences were caused by nonspecific protein adsorption in saliva. Reproduced with permission from [113]. Copyright 2020 The Royal Society of Chemistry.

The development of specific probes can effectively detect and quantify the specific protein content in body fluids, which plays an important role in drug effectiveness evaluation and disease diagnosis [111]. However, in the actual application environment, both label-free and label-assisted technology are inevitably affected by biofouling [112]. For the label-free surface plasmon resonance (SPR) probe and quartz crystal microbalance (QCM) probe, the change of measured value can accurately reflect the interaction between molecules. Therefore, in specific antigen recognition applications, any nonspecific adsorption behavior on the surface of the label-free probe will also be recorded as noise, thus reducing the sensitivity and detection limit of the label-free probe [112]. Zwitterionic polymer brush is the main way to provide antifouling performance for probes, but the structure of the polymer brush also has some defects. On the one hand, if the surface of the probe is modified by antibody/active functional group first, and then zwitterionic polymer brushes are prepared, the interaction between molecules and active sites will be disturbed by the steric hindrance of polymer brushes [113,114,115]. On the other hand, if antifouling zwitterionic polymer brushes are prepared first and then modified with antibody/active functional groups, active sites that can bind antibody/active functional groups must be provided on the polymer side-chains, which will greatly limit the types and applications of zwitterionic polymer brushes [112]. In order to expand the application of zwitterionic materials in the field of probes, the interpenetrating polymer network hydrogel (IPN) combined with surface-initiated atom transfer radical polymerization (SI-ATRP) prepared polymer brushes and active site hydrogel thin films maintained the recognition sensitivity of recognition molecules with the antifouling property. Zhang et al. [113] used SI-ATRP to prepare pSBMA polymer brushes on the QCM probe surface and interpenetrated them with phenylboronic acid (PBA)-PAAm hydrogels. The thickness of IPN hydrogel was controlled by pSBMA polymer brushes. The study confirmed that the IPN hydrogel with a thickness of 50 nm exhibited an excellent antifouling effect and was able to effectively detect glucose in saliva with a concentration of 0–50 mg/L (Figure 13).

For label-assisted encoding hydrogel probes, biofouling will also become an important problem, limiting its application in complex environments [116]. Currently, widely used PEG-encoding hydrogel probes are trapped in coagulation problems in the whole blood environment and cannot provide accurate visualization information [117]. Using MPC as the monomer, Roh et al. prepared the encoding hydrogel through degassed molding lithography (DML) and then used Michael addition of the thiol group (-SH) and unreacted methacrylic acid functional group to achieve antibody immobilization. PMPC-encoding hydrogel not only successfully achieved multiple immunoassays but also enhanced the sensitivity of probes because of the improvement of antifouling performance. In platelet-rich plasma, PMPC-encoding hydrogel increased the antifouling effect by 70%, which made PMPC-encoding hydrogel promising to be applied in complex clinical conditions [116].

### 4.4. Implants

Implantable devices have a very wide application prospect in clinical treatment. However, thrombosis, infection and foreign body response (FBR) are always of concern to clinicians [118].

**Figure 14:**
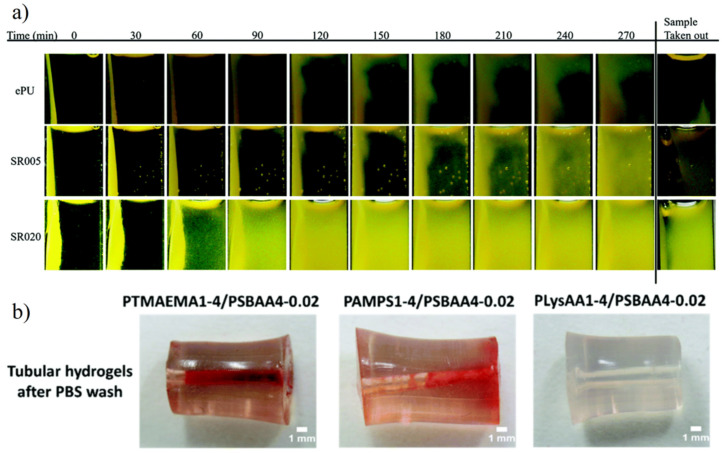
Anticoagulation performance of a zwitterionic hydrogel. (**a**) Time-lapse image of coagulation showed no interaction between electrospun reinforced zwitterionic hydrogel and fibrin network. Reproduced with permission from [50]. Copyright 2020 The Royal Society of Chemistry. (**b**) Anticoagulation and antithrombosis of tubular pLysAA/SBAA double network hydrogel. Reproduced with permission from [6]. Copyright 2020 The Royal Society of Chemistry.

Due to the adhesion of platelets on the surface of materials, coagulation and thrombus formation immediately occur, and blood contact implants often face the problem of pore blockage. Recently, polytetrafluoroethylene (ePTFE) small caliber artificial blood vessels (based on fouling release) still face blockage caused by coagulation in the process of long-term use [119]. Zwitterionic materials showed excellent anti-platelets adhesion and biocompatibility. Liu et al. [50] prepared electrospun reinforced SBMA hydrogel with high tensile strength and excellent anti-platelets adhesion. Time-lapse image of coagulation even showed there is no interaction between composite hydrogel and fibrin network during the whole coagulation period (Figure 14a). Huang et al. [6] prepared poly(lysine acrylamide) (PLysAA)/poly(sulfobetaine acrylamide) (PSBAA) double network zwitterionic hydrogel. The prepared tubular hydrogels with an inner diameter of 1 nm showed no red blood cells (RBCs) or blood clotting were found after 2 h human whole-blood circulation with an average flow rate of 20 mL/min at 37 °C (Figure 14b).

Unlike the reusable and washable medical apparatus mentioned above, implantable medical devices, such as central venous catheters (CVC), have been used in the physiological environment for a long time [118]. At present, there is still a 5–15% infection rate in the use of cardiovascular medical devices [120]. These infections can further lead to life-threatening complications and a significant burden on the health care system. Although antibiotic coated materials can reduce infection, they can only be used in short-term implanted medical devices [121]. As mentioned above, zwitterionic hydrogels, though lacking in bactericidal capacity, can prevent bacterial adhesion. Koc et al. [122] designed various sulfobetaine hydrogel coatings on glass substrates, showing excellent resistance to microorganisms (2–8% related to butyl methacrylate (BMA) hydrogel coating) in severe conditions. Liu et al. [123] prepared anti-bacterial hydrogel with copolymerization of sulfonated quaternization of 1-vinylimidazole (VIm) based zwitterions (VImSL) and [3(methacryloylamino)propyl]trimethyl ammonium chloride (MPTAmC). Results showed excellent anti-bacterial activities against both *E. coli* and *S. aureus*. Sheng et al. [124] designed a multifunctional peptide (BrEK) with antimicrobial sequence BF2b, a gelatinase B responsive linker and a zwitterionic EK block. This strategy also provides excellent antibiotic capacity and anti-bacterial adhesion as coating.

The inevitable foreign body response (FBR) leads to serious challenges to the performance of implant devices due to the formation of a collagenous capsule around the implants [2]. To avoid FBR in clinical application and enhance the comfort of patients, biologically related materials need to move closer to the direction of bionics. Hydrogel materials similar to human tissue structure have tended to meet functional requirements in performance, but the relatively high biocompatibility still cannot overcome FBR in clinical applications [125]. As mentioned above, materials not to trigger FBR and further fibrous capsule formation after long-term implantation are important in biological engineering [126]. PEG and PHEMA hydrogels, the earlier low fouling hydrogel implants, have confirmed that the performance of antifouling determines the compatibility of synthetic polymer implants in vivo [127,128,129].

**Figure 15:**
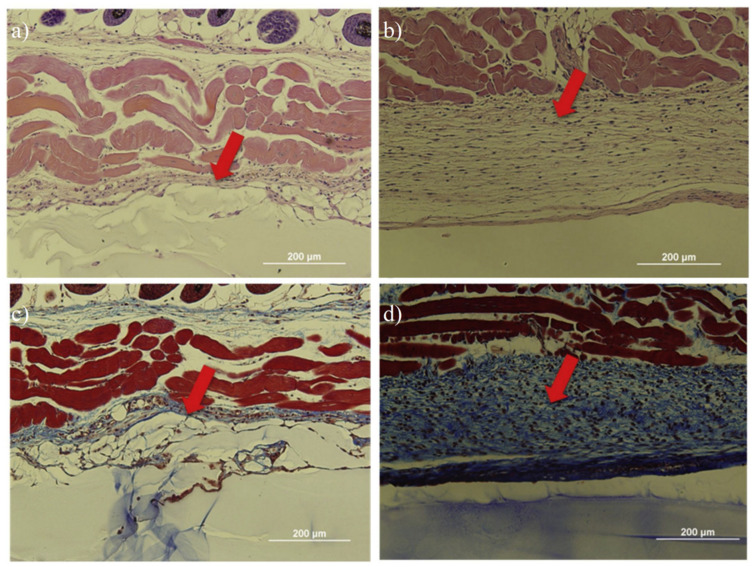
Zwitterionic methacryloyl-L-lysine imparted hydrogel showed less negligible inflammatory response and foreign body reaction after 2 months of implantation in mice. (**a**,**b**) H&E staining for skin tissues in contact with the zwitterionic hydrogel and pHEMA hydrogels. Less immune cells were observed around zwitterionic hydrogel (**a**) as compared to pHEMA hydrogel (**b**). (**c**,**d**) Masson’s trichrome staining for skin tissues with zwitterionic hydrogels and pHEMA hydrogel. Less fibrin capsule formation was observed around zwitterionic hydrogel (**c**) while much thicker and dense fibrin formation was observed around pHEMA hydrogel (**d**). Reproduced with permission from [13]. Copyright 2020 Elsevier Ltd. All rights reserved.

Chan et al. [13] used amino acid-terminated methacryloyl-L-lysine (MLL) to impart zwitterionic property to the calcium crosslinked hydrogels. Results showed high resistance to the formation of fibrous capsules for at least two months when subcutaneously implanted in mice (Figure 15). Zhang et al. [14] prepared zwitterionic pCB hydrogels to evaluate the inflammatory response to the implants one week after implantation and capsule formation after four weeks. Results showed prepared few inflammatory cells were detected at the tissue-PCBMA hydrogel interface while numerous inflammatory cells were observed at the tissue-PHEMA surface. Fibrous capsule resistance of pCB hydrogel sustained for three months while PHEMA hydrogel trigger FBR and fibrous capsule formation within one month. Dong et al. [47] prepared zwitterionic elastomeric network (ZEN) by interpenetrating pCB and pSB network to achieve both high strength and one-year fibrous capsule resistance.

For special conditions, the demand for functional implants will not be limited to avoiding FBR. For example, artificial cartilage-like implants should not only avoid FBR but also have excellent lubricity and mechanical strength to meet the requirements of daily activities. Bonyadi et al. [130] prepared four double network hydrogels using poly(2-acrylamido-2- methyl propanesulfonic acid) (PAMPS) as the first network and P(NIPAM (90%)-co-different charged monomers (10%)) as the second network. Results showed that the introduction of only 10% zwitterionic copolymers decreased the surface friction of hydrogel significantly, and the FBS interacted with hydrogel and cartilage in a similar manner. Milner et al. [131] prepared PAMPS-PAAm-PMPC triple-network hydrogels for partial joint repair. Among them, the PAMPS-PAAm double-network provides mechanical properties, and the PMPC network provides wear-resistant friction properties. Ren et al. [132] prepared xanthan gum-zwitterionic nanogel additives that can bind to collagen II on damaged cartilage. The prepared nanogel has minimally invasive or injection potential to alleviate abnormal symptoms caused by insufficient lubrication of damaged joints. The introduction of xanthan gum acted reactive oxygen species (ROS) scavenger while collagen II binding peptide WYRGRL immobilized nanogels on the surface of the cartilage. In the in vitro model, low friction coefficient (<0.1) and high ROS scavenging (36.89% for 2,2-diphenyl-1-picrylhydrazyl (DPPH), 62.5% for OH, 74.85% for O_2_^−^ and 82.6% for H_2_O_2_) were well presented.

### 4.5. Biosensor

Hydrogel biosensors are of great importance in monitoring the physiological status and disease control of patients. Sensitivity, reliability, and repeatability are the basis of biosensor design [133]. For hydrogel biosensors, the ionic conductivity can influence the sensitivity of biosensors, and the ability to resist biofouling determines the reliability of equipment during long-term use, and flexibility and self-healing ability affect the repeated response capability of the device [134].

To improve the sensitivity of hydrogel sensors, it is possible to introduce high conductivity nanomaterials [135,136] or conductive polymers [137,138]. However, the introduction of nanomaterials will inevitably lead to heterogeneity, which will damage the stress response, repeatability, and stability; The hydrogel sensors prepared by conducting polymers lack sufficient mechanical strength and fatigue resistance [59].

**Figure 16:**
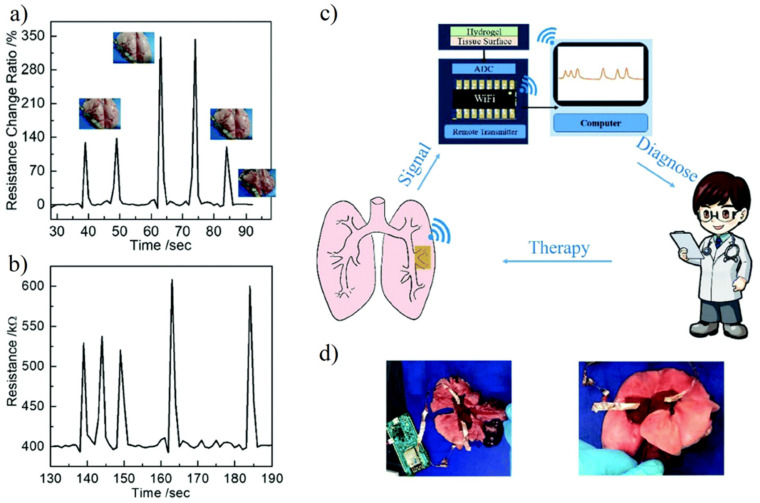
Hydrogel sensors for real-time monitoring and simulated lung breathing. (**a**,**b**) Wired transmission (**a**) and wireless transmission (**b**) of the resistance from simulated lung breathing. No significant signal differences were collected in wired or wireless transmission in term of sensitivity. (**c**) Schematic illustration of wireless monitoring of lung breathing. (**d**) Images of rabbit lungs with wired (left) or wireless (right) transmission hydrogel sensor. Reproduced with permission from [63]. Copyright 2020 The Royal Society of Chemistry.

Zwitterionic hydrogel has ionic conductivity, biofouling resistance, and self-healing ability. It is an ideal material for hydrogel biosensors. Zhang et al. [59] prepared P (HEAA-co-SBAA) and PEDOT:PSS interpenetrating network hydrogels. The IPN network hydrogel has an excellent antifouling capability, high tensile strain (4000–5000%) and strength (0.5 MPa), and also shows good self-healing ability. The synergy of the zwitterionic network and conductive polymer also enhances the sensitivity of the device. Another factor affecting biosensors is the fit between devices and tissues. Using the suture method to fix the equipment at the specified position will not only cause additional damage but also have defects in the fitting [139]. In order to better detect the target tissue in real-time, the hydrogel biosensor needs better fixation methods to reduce the loss of signal transmission [140]. Pei et al. [63] introduced dopamine-modified Laponite XLG into the pSBMA polymer network. The synergistic effect of catechol groups and zwitterionic dipole–dipole interaction provides excellent tissue adhesion for the device. Results showed that the prepared hydrogel biosensor had 0.0215 S/m ionic conductivity and high strain sensitivity (gauge factor GF = 4.3, 0–670% strain). The efficacy of wireless transmission of the collected signals from simulated dynamic lung and heart motions to the computer for diagnosis was also demonstrated (Figure 16).

### 4.6. Drug Delivery

Hydrogel in the nanoscale (i.e., nanogel) is an important carrier for both small molecules and macromolecular pharmaceuticals delivery in vivo due to superior colloid stability and flexibility in controlling the release of drugs [141]. However, in practical research, whether nanocarriers can produce enough effect is seriously determined by the biological immune system. Targeted binding and metabolic clearance have become a racing relationship [142]. Among them, the rate of metabolic clearance is often greater than the ability of targeted binding. For example, nanodrugs have less than 1 h circulation half-life without PEG [143]. The recognition of nanocarriers by the reticuloendothelial system is an important factor to determine whether they will be metabolized rapidly [144].

PEG-based nanogel showed both temperature responsibility and a long circulation life due to the antifouling characteristic [145]. However, there is still interaction between PEG and protein, especially in complex biological environments (such as whole plasma environment) [11], and the PEG antibodies found in recent years also show that the application of PEGylated drugs will be limited [146]. Realizing true non-fouling is the key point to avoid reticuloendothelial system recognition and achieve real long blood circulation [142].

Zwitterionic nanomaterials have been proved to contribute to the long-term efficacy of drugs. A very strong example is the relief and life-saving of organophosphorus (OP) poisoning. Zhang et al. [147] used CBAA nanogels to encapsulate organophosphorus hydrolase (OPH). The half-life of OPH in rats was extended from 0.43 h to 26.2 h, and the OPH in plasma could still be detected after 72 h. In repeated dosing trials, nanogel-encapsulated OPH avoided the accelerated blood clearance (ABC) phenomenon when OPH-only was injected repeatedly. In addition, CBAA nanoparticles did not induce the production of corresponding polymer antibodies in either subcutaneous or intravenous, which had better safety than PEG materials. When the effective dose of 1 mg/kg nanogel-loaded OPH was injected in advance, the poisoning caused by organophosphate could be prevented in 6 d, while 3 h could only be maintained at the same dose of OPH alone.

**Figure 17:**
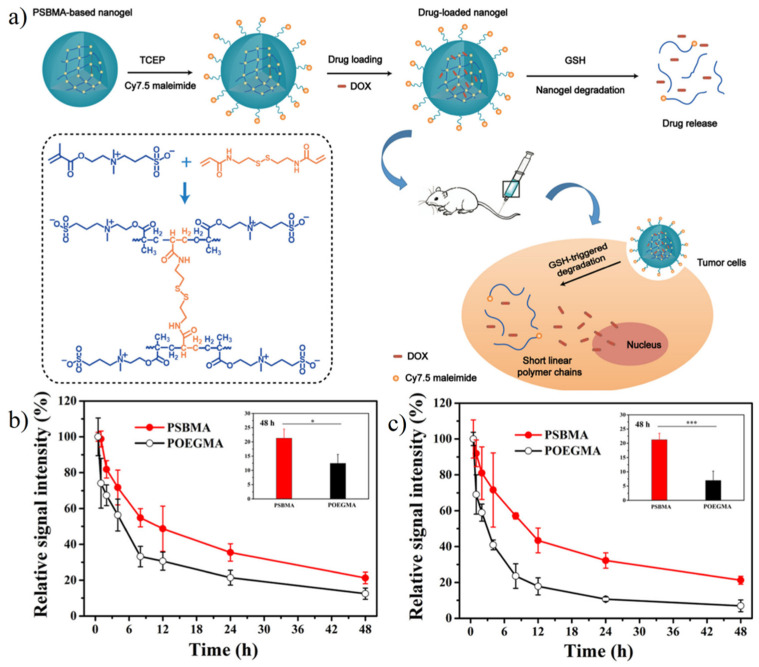
Redox-responsive controlled release of pSBMA-based nanogel. (**a**) Schematic illustration of doxorubicin-loaded biodegradable pSBMA-based nanogel for tumor therapy. (**b**) Blood retention of POEGMA nanogel and PSBMA nanogel after 1st injection. * < 0.05. (**c**) Blood retention of POEGMA nanogel and PSBMA after 2nd injection. *** < 0.00001. There was no significant difference of pharmacokinetics between 1st and 2nd injection shown in the PSBMA nanogel group. Reproduced with permission from [149]. Copyright 2018, American Chemical Society.

Zwitterionic nanogel is also suitable for the delivery of targeted tumor drugs. Lu et al. [148] prepared hydrazone crosslinked zwitterionic poly (aspartic acid) nanogel with Adriamycin (DOX) loaded. Hydrazone acted as a pH-trigger for drug release. Results showed more than 70% DOX was released in 12 h at pH 5.0, while less than 25% DOX was released at pH 7.4 in the same period. Men et al. [149] prepared redox-responsive zwitterionic SBMA nanogel with glutathione (GSH) sensitive disulfide bond contained crosslinker. Results showed more than 85% DOX release over 12 h in reductive condition (10 mM GSH) while less than 7% without GSH. Moreover, pSBMA nanogel showed long circulation with 21.3% retained in blood at 48 h, while pOEGMA nanogel showed only 12.5%. The second injection after 7 d demonstrated the better biocompatibility of SBMA with similar pharmacokinetics behaviors over 48 h while drastically reduced blood retention (6.9%) of POEGMA nanogels was observed (Figure 17). She et al. [150] prepared hypoxia-degradable zwitterionic phosphorylcholine nanogel with the azobenzene-based crosslinker. Results showed a significantly stronger glioblastoma inhibition effect and notably improved the increase in survival time.

Nanogels prepared by inverse emulsion polymerization require strictly removal of large amounts of surfactant, which limits further application. Ekkelenkamp et al. [151] prepared zwitterionic poly(amidoamine)s (PAAs) nanogel by solution displacement, which was also called inverse nanoprecipitation. In order to further simplify the process, Men et al. [149] used reflux precipitation to prepare nanogel at 100 °C. Both methods solved the excessive surfactant residual in the finish products, which may promote the clinical application of a zwitterionic nanogel controlled release system.

### 4.7. Cell Capsulation

Cellular therapy has profound significance in the treatment of chronic diseases [152]. However, the immune system response caused by allogeneic materials leads to the formation of the fibrous capsules at the implantation site, which cuts off the supply of nutrients and eventually leads to cell death [153]. Although the application of immunosuppressants can prolong the life of implanted cells to a certain extent, it also has obvious side effects [154]. Chitosan/alginate multilayers with zwitterionic phosphorylcholine-modified chondroitin-4-sulfate deposition had proven the efficacy to protect implanted insulin-producing pancreatic β-cell spheroids [155]. However, the structure, morphology, and stability of encapsulated carriers need to further improve the survival and function of islets. Zwitterionic hydrogels are extremely suitable as cell-capsulated systems due to their extraordinary antifouling characteristic.

**Figure 18:**
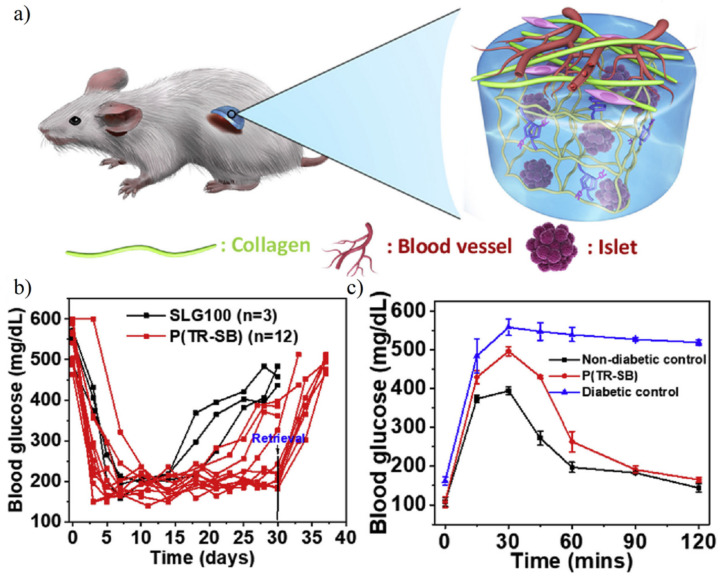
Islet encapsulation for diabetes treatments using triazole-zwitterionic hydrogel. (**a**) Schematic illustration of low fibrosis and high vascularization around islet encapsulated hydrogel. (**b**) Blood glucose concentration after 30 d of implantation. (**c**) Islet implanted mice showed normal blood glucose in 30 days and the ability to metabolize intraperitoneal glucose within 90 min was restored. Reproduced with permission from [57]. Copyright 2019 Elsevier Ltd. All rights reserved.

Liu et al. [57] prepared triazole–zwitterionic hydrogel to fulfill mechanical strength requirements and islet capsulation. Results showed the islet implanted mice maintain the normal blood glucose level in 30 d. Further intraperitoneal glucose tolerance test confirmed islet implanted mice restore normoglycemia with 90 min, which was similar to normal mice (Figure 18). Zwitterionic SB- or CB-modified nature polysaccharides such as alginate hydrogel were also developed in cell capsulation. After long-term 200 d transplantation, diabetic mice still maintained normoglycemia [156].

### 4.8. Cell Culture

Hydrogel is suitable for the 2D and 3D cultures of cells. Compared with conventional cell culture medium, hydrogel 2D culture can control environmental factors, such as stiffness and ligands, more accurately, which may be quite useful for understanding specific behaviors in the cell life cycle [157]. Cells are less constrained in a 2D environment than a 3D medium, while hydrogel as a 3D culture medium can obtain better cell state and biological activity, which is very important for the development of regenerative medicine [157].

The multipotency of stem cells is a very important research content of regenerative medicine, which makes the 3D culture technology of stem cells receive extensive attention [158]. However, stem cells are difficult to maintain their multipotency in vitro and are prone to differentiation induced by culture medium, such as adipocytes in low modulus environments and osteoblasts in high modulus environments [159]. Therefore, in order to maintain the multipotency of stem cells, it is often necessary to add chemical inhibitors [160]. Through accurate control of chemical and physical factors, high molecular weight PEG hydrogel has been able to maintain the multipotent nature of stem cells [161], but unfortunately, this control condition is too harsh, so it is difficult to achieve consistent promotion in clinical applications [159]. On the other hand, an interesting study has confirmed that the multipotency maintenance of stem cells is closely related to the nonspecific protein adsorption in the culture medium [162].

**Figure 19:**
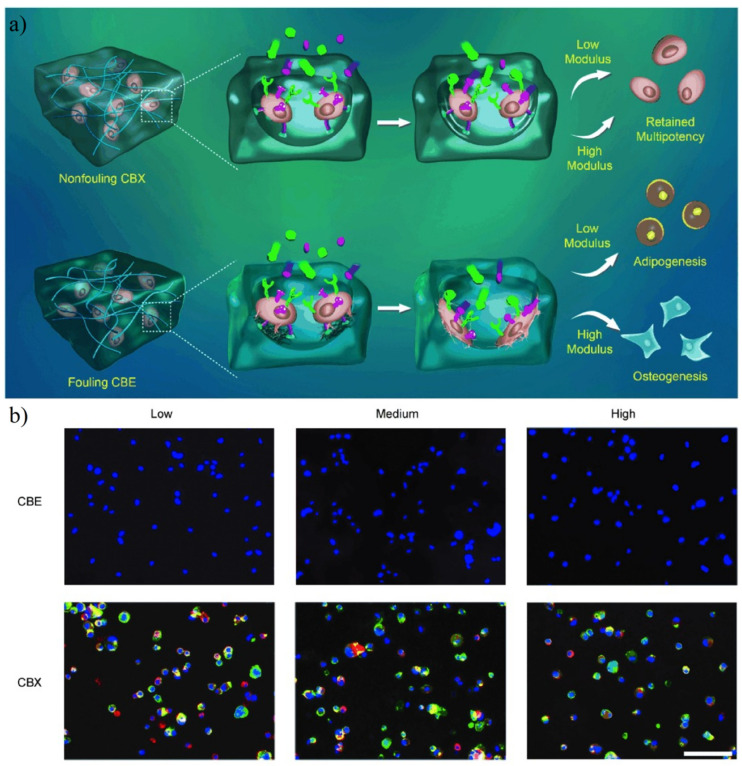
Fate choice of hMSCs in antifouling PCBAA hydrogel with zwitterionic crosslinker (named as CBX) and non-ionic crosslinker (named as CBE). (**a**) Schematic illustration of the behavior of hMSCs in CBX and CBE. cRGD moieties are denoted as cyan hemispheres, and differentiation factors are denoted as colored particles. (**b**) Immunofluorescence staining of hMSCs by undifferentiated-cell markers ALCAM (green) and STRO-1 (red; nuclei: blue) in CBX and CBE. Most of the hMSCs in hydrogel CBE were not able to express this biomarker. Reproduced with permission from [159]. Copyright 2014 WILEY-VCH Verlag GmbH & Co. KGaA, Weinheim.

The zwitterionic hydrogel can maintain the multipotency of stem cells in a wider physical condition. Bai et al. prepared human mesenchymal stem cell (hMSCs) encapsulated zwitterionic PCBAA hydrogel with a zwitterionic crosslinker carboxybetaine dimethacrylate (named as CBX) and non-ionic crosslinker ethylene glycol dimethacrylate (named as CBE). Results showed that hMSCs in hydrogel CBX were positive for stem cell biomarker STRO-1 and ALCAM, while most of the hMSCs in hydrogel CBE were not able to express these biomarkers (Figure 19). Further, Bai et al. [37] prepared poly(carboxybetaine)-based hydrogels (ZTG) with strain-promoted azide-alkyne cycloaddition (SPAAC) ‘click’ reaction to capsulate hematopoietic stem and progenitor cells (HSPCs). Results showed ZTG inhibited ROS production (an important factor to trigger HSPCs differentiation) and ROS-related pathways in HSPCs culture. For both primitive and adult HSPCs, ZTG culture both promoted the significant expansion of the HSPCs population without differentiation. Zhang et al. [36] prepared antifouling hydrogel with methacrylated hyaluronic acid (HAGMA) and a copolymer of CBMA and *N*,*N*′-bis(acryloyl)cystamine (BAC) via thiol-ene click chemistry. The encapsulated human mesenchymal stem cells (hMSCs) remained primitive metabolically active.

### 4.9. Wound Healing

The process of wound healing requires the harmonious interaction between cells, growth factors and extracellular matrix (ECM) protein [163]. In the process of wound healing, keeping the wound moist and controlling the inflammatory response are the most important factors. The former can promote the transfer of substances in the wound area, while the latter plays an important role in the release of related biochemical substances [164,165]. The results show that excessive inflammation will lead to the loss of skin function and appendages and hinder wound repair [166]. In the current wound healing materials, both hydrogel and hydrocolloid materials will inevitably cause more inflammatory responses due to the recognition of the immune system [167]. Even the low fouling capacity of PEG hydrogel is also unable to avoid immune system recognition in the complex wound environment [167]. In addition, PEG is susceptible to oxidation, which will lead to the aggravation of inflammation and is not conducive to long-term wound healing [168].

**Figure 20:**
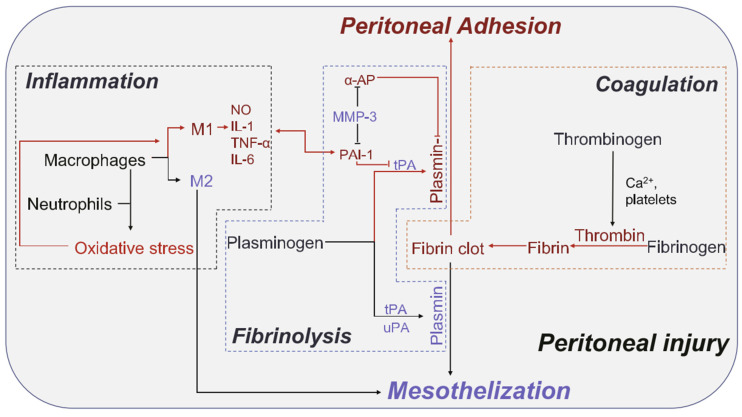
Schematic diagram of the interactions among coagulation, inflammation, and fibrinolysis system. Reproduced with permission from [170]. Copyright 2020 Acta Materialia Inc. Published by Elsevier Ltd. All rights reserved.

For zwitterionic hydrogel, the current research not only confirms that it can evade the recognition of the immune system but also maintain structural integrity in the long-term subcutaneous implantation and does not produce fibrous capsules [47]. The zwitterionic hydrogel also shows the control of ROS in the wound area so that macrophage type M1 with a high inflammatory response can transform to M2 with the lower inflammatory response (Figure 20) [169,170]. The latter appears more in the behavior of tissue regeneration and promotes the generation and recovery of the tissue functional structure.

**Figure 21:**
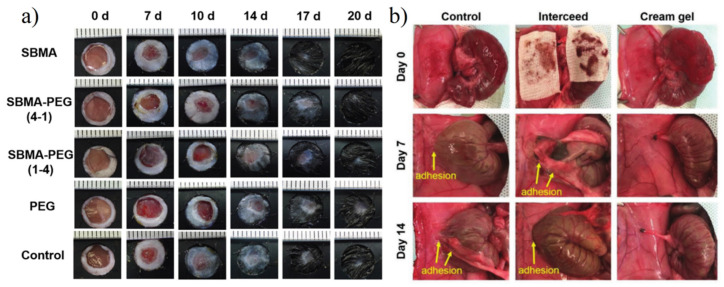
Typical wound healing effects of the zwitterionic hydrogel. (**a**) Wound healing performance of zwitterionic hydrogel applied on healthy mice with back circumcision. Zwitterionic hydrogel showed both a much faster recovery rate and better hair growth. Reproduced with permission from [167]. Copyright 2018 Elsevier Ltd. (**b**) PCBAA cream-gel with biodegradable BAC crosslinker for peritoneal adhesion. On days 7 and 14 post-surgery, adhesions were observed in the untreated control and intercede film groups, while no adhesion was observed in rats treated with cream-gel. Reproduced with permission from [171]. Copyright 2020 Wiley-VCH GmbH.

Zwitterionic hydrogels have been widely used in both inside and outside wound healing of the human body. For outside wounds, Wu et al. [167] evaluated wound healing efficacy between the zwitterionic hydrogel and non-ionic PEG hydrogel. Zwitterionic hydrogel showed both much faster recovery rate and better hair growth (Figure 21a). For inside wound or potential postoperative adhesion, Zhang et al. [171] prepared pCBAA cream-gel with a biodegradable BAC crosslinker to further improve resistant peritoneal adhesion (Figure 21b).

For better wound healing effects, the corporation of zwitterionic hydrogel and assistant factors such as growth factors, antimicrobial agents, and antioxidants are widely used in recent wound healing research. Qiu et al. [172] modified dextran with SB unit or CB unit and then formed SB-Dextran and CB-Dextran hydrogel with the borax crosslinker. The anomeric hydrogens of α-D-glucopyranose can be attacked by free radicals easily, which provide dextran scavenging capacity against the hydroxyl radical. Dai et al. [66] prepared zwitterionic sulfobetaine acrylamide hydrogel incorporated with laponite nanoplatelets, curcumin and methacrylamide dopamine (DMA). Among them, hydrophobic curcumin is immobilized on laponites and then provides antimicrobial activities. DMA provides better adhesion to the wound area. Huang et al. [173] prepared SBAA hydrogels with hectorite and Ag nanoparticles for infected chronic wound healing. Results showed significant improvement on infected dorsal wounds on rats with induction of diabetes by Streptozotocin compared to commercial AQUACEL^®^ Ag dressing (ConvaTec, UK). Xiao et al. [174] prepared fibroblast growth factor-2 (FGF-2)-loaded SBMA hydrogel and then compared the in vivo wound healing and in vitro cell proliferation with FGF-2-loaded PEG hydrogel. The FGF-2 release was prolonged in SBMA hydrogel compared with PEG hydrogel. In both in vitro cell proliferation and in vivo wound healing tests, FGF-2-loaded SBMA hydrogel showed the best effects.

## 5. Conclusions and Perspectives

Zwitterionic hydrogels have already been involved in much more complex biomedical application scenarios. In this review, we have summarized the unique features of zwitterionic hydrogels, including antifouling properties, excellent biocompatibility, anti-polyelectrolyte effect, high ion conductivity, self-healing, and super-lubrication, and the corresponding biomedical applications. Zwitterionic hydrogels have demonstrated the potentials to substitute widely used PHEMA and PEG hydrogel in various applications. Lots of efforts have been performed to adjust these characteristics for further application. However, there is still much work to be implemented to realize the full potential of zwitterionic hydrogels. First, apart from antifouling and those unique properties mentioned above, other interesting properties, such as pH responsivity, thermal responsivity, should be explored, which might be useful in drug delivery, tissue regeneration, lubrication and biosensing. Second, most of the studies discussed in this review rely on the in vitro or small animal models. Large animal studies and clinical trials with long-term biological responses should be processed in order to receive a real product.

## Figures and Tables

**Table 1:** SR_equibrium_ of zwitterionic copolymer hydrogels in water and NaCl solution (1 mol/kg) at room temperature. Reproduced from [49]. Copyright 2019, American Chemical Society.

Sample	Sr_equibrium, Water_	SR_equibrium, NaCl_	SR_equibrium, NaCl_/SR_equibrium, Water_
PNaSS	18.68	6.21	0.33
PSBMA1NaSS0.5	18.22	7.51	0.41
PSBMA1NaSS1	33.27	10.22	0.30
PSBMA1NaSS2	71.94	16.39	0.23
